# Higher BNP levels within physiological range correlate with beneficial nonfasting lipid profiles in the elderly: a cross-sectional study

**DOI:** 10.1186/s12944-015-0168-1

**Published:** 2016-01-05

**Authors:** Wen-Tao He, Masayuki Mori, Xue-Feng Yu, Tsugiyasu Kanda

**Affiliations:** Department of Community Medicine, Kanazawa Medical University Himi Municipal Hospital, Himi, Toyama 935-8531 Japan; Department of Endocrinology, Tongji Hospital, Tongji Medical College, Huazhong University of Science and Technology, Wuhan, 430030 China

**Keywords:** BNP, Nonfasting lipid profiles, The elderly, Dyslipidemia

## Abstract

**Background:**

Emerging studies indicate that B-type natriuretic peptide (BNP), a well-known biomarker for heart failure, also plays pivotal roles in metabolic control. Circulating BNP levels progressively increase as ages grow older. However, the association between BNP levels and lipid metabolism in the elderly remains unknown.

**Methods:**

A total of 680 eligible volunteers (male/female: 334/346) aged between 60 and 80 years old without overt heart failure (BNP <100 pg/ml) were enrolled. Random nonfasting venous samples were obtained for biochemical analysis. The subjects were stratified based on BNP quartiles: BNP Q1 (range: 2.2–9.0 pg/ml), Q2 (9.1–20.4 pg/ml), Q3 (20.5–44.4 pg/ml) and Q4 (44.6–99.7 pg/ml). Difference of metabolic parameters was compared among the subjects grouped by BNP quartiles. Univariate correlation and multiple linear regression were performed to analyze the association between BNP levels and metabolic parameters. The odds ratios (OR) and 95 % confidence intervals (CI) for dyslipidemia in subjects within BNP Q1-3 relative to subjects within BNP Q4 were calculated.

**Results:**

Circulating BNP levels positively correlated with age, while negatively correlated with body mass index (BMI), eGFR and non-HDL. Subjects with lower BNP quartiles had significantly elevated prevalence of dyslipidemia, including hypertriglyceridemia, hyper-LDL-emia and hypercholesterolemia. The OR of hypertriglyceridemia and hypercholesterolemia for subjects within BNP Q1-2 significantly increased relative to BNP Q4.

**Conclusions:**

The elderly people with higher BNP levels have significantly reduced risks for nonfasting dyslipidemia. Verification of the cause-effect relationship between BNP and dyslipidemia may bring therapeutic implications.

## Background

Natriuretic peptides (NPs) are a group of functionally and structurally related peptides, including atrial NP (ANP), B-type NP (BNP) and C-type NP (CNP). BNP is predominantly expressed by ventricular cells and correlates better with the severity of heart failure than ANP, so BNP has gained more intense researches as a clinically useful biomarker [1, 2].

Although higher BNP levels are often associated with adverse cardiovascular outcomes [3, 4], growing evidence has demonstrated that elevated BNP plays beneficial roles in metabolic regulation under non-heart failure settings. As opposed to cardiac cachexia partly mediated by raised BNP levels [5, 6], the inverse correlation between BNP and body mass index (BMI) has been consistently reported in a number of observational studies [7–9]. Mechanistically, BNP directly promotes mitochondrial biogenesis in skeletal muscles and white adipocytes to enhance energy expenditure [10, 11]. Through these mechanisms, transgenic mice overexpressed with BNP are highly resistant to high-fat diet induced obesity, insulin resistance and diabetes [10]. Thus, heart-derived BNP has been viewed as a central player in the control of body weight and energy metabolism.

Recently, it has been demonstrated that higher BNP levels correlate with favorable adiposity profiles, including reduced deposition of visceral and liver fat [12]. The pattern of body fat distribution is an independent factor associated with metabolic syndrome in the elderly population, including dyslipidemia [13]. Therefore, BNP might affect the lipid metabolism. Schlueter N et al. have postulated that BNP holds the potential to treat a series of metabolic disorders [14]. Paradoxically, existing data also suggest that higher BNP levels provide independently predictive value on assessment of future cardiovascular disease (CVD) risk [3], whereas cholesterol-lowering rosuvastatin treatment effectively improves the adverse outcome. In another study, a nonlinear U relationship between BNP and metabolic syndrome has been observed [15]. It seems that the influence of BNP on CVD risks in clinic is far more complicated than experimental results from transgenic murine models. However, overexpression of BNP under cardiac stress belongs *per se* to a cluster of protectively compensatory events. Consequently, BNP to a certain extent benefits cardiac function through generalized loss of fat tissue in addition to natriuresis. Thus, high BNP levels might be associated with lipid metabolism. Nevertheless, the direct data available on this issue remain lacking.

We hypothesized that BNP might play beneficial roles in lipid metabolism. To this end, we investigated the associations between BNP levels and nonfasting lipid levels in the elderly people without overt heart failure. We found that the elderly people with higher BNP levels tended to have reduced prevalence of hypertriglyceridemia, hypercholesterolemia and hyper-LDL-emia, which supports the beneficial roles of BNP in lipid metabolism. This study was performed based on two major respects. Firstly, the elderly people have larger variation range of BNP levels than younger people [16], which might make the difference in lipid metabolism more visible. Secondly, emerging evidence supports the notion that nonfasting lipid levels provide more predictive values on risks for CVD than fasting lipids in additional to increased patient compliance. In several large population-based prospectively observational studies, nonfasting triglyceride (TAG) has been shown to be a superior risk predictor for CVD compared with fasting TAG [17, 18]. Nonfasting total cholesterol (TC), low-density lipoprotein cholesterol (LDL-C) and high-density lipoprotein cholesterol (HDL-C) change minimally in response to normal food intake [19].

## Methods

### Study subjects

All subjects who participated in the cross-sectional research were from community-dwelling residents received annual examinations in Himi Municipal Hospital, Kanazawa Medical University. From Jan 2012 to May 2015, a total of 1248 volunteers who met the following criteria were enrolled in the study. The inclusion criteria included: (1) age above 60 and less than 80 years old; (2) serum Cr levels less than 2 mg/dL; (3) no overt dysfunction of heart, liver or lung diseases; (4) the level of BNP between 0 and 100 pg/mL; (5) 0.1 mIU/L ≤ TSH ≤10 mIU/L, FT3 and FT4 within the normal range. As various factors affect the levels of BNP and lipids, such as acute inflammation, injury, thyroid hormones, blood volume and sex steroids, we excluded those who met the exclusion criteria, including: (1) acute infection and stress in the last three months (*n* = 59); (2) medication on lipid-lowering drugs, glucocortoids, sex steroids, diuretics and thyroid-related drugs (*n* = 413); (3) overt hyperthyroidism or hypothyroidism (*n* = 96). The remaining 680 participants were involved in this study. Electronic data on medical history for each participant are accessible in the computerized database of Himi Municipal Hospital. All participants provided informed written consent. The protocols of the study were approved by the Ethics Board of Kanazawa Medical University Himi Municipal Hospital (protocol number 34).

### Anthropometric measurements

Anthropometric parameters obtained in this study included weight, height and BMI. BMI was calculated as body weight in kilograms divided by the height squared (kg/m^2^).

### Biochemical investigations

Random venous blood samples were collected in tubes containing liquid EDTA and were centrifuged to obtain plasma for further biochemical analysis. Liver and kidney function tests, UA, TC, HDL-C, LDL-C and TAG were measured by automated analyzer (JCA-BM6050 Bio Majesty, Nihon Denshi Co. Ltd., Tokyo, Japan). The minimal detectable limit of BNP was 2.0 pg/mL. The mean intra-assay and inter-assay coefficients were 2.1 and 3.6 %, respectively. Non-HDL was defined by the formula: non-HDL = TC − HDL-C. HbA1c was examined by a latex coagulation method. BNP levels were measured using chemiluminescent enzyme immunoassay kit (MI02 Shionogi BNP; Shionogi Co. Ltd., Osaka, Japan). The serum thyroid function was measured by chemiluminescence assay. The eGFR was calculated by an equation for Japanese: eGFR (ml/min/1.73 m^2^) = 194 × Cr^-1.094^ × age^-0.287^ × 0.739 (if female). Currently, there are no available age- and population-based standards for random lipid levels. Previous research has found that minimal difference exists between nonfasting and fasting lipid levels [19]. In this study, definition criteria for abnormal lipid metabolic indicators were used with reference to the following statement or guideline, including American Heart Association (AHA) scientific statement on triglycerides and CVD [17], and American Association of Clinical Endocrinologists’ Guidelines for Management of Dyslipidemia and Prevention of Atherosclerosis (2012) [20]. Specifically, cut-off points for dyslipidemia were defined as any one of the following: TC ≥200 mg/dL, LDL-C ≥130 mg/dL, HDL-C ≤60 mg/dL or TAG ≥200 mg/dL.

### Statistical analysis

The study population was separated into four groups according to BNP quartiles, namely BNP Q1-Q4. The normality of distribution was performed by Kolmogorov-Smirnov test. Variables with skewed distribution were expressed as median and quartile ranges (25th to 75th percentiles). Continuous variables with normal distribution were expressed as mean ± standard deviation (SD). Categorical variables were expressed as numbers and percentages. Chi-square test was used to compare categorical variables. The difference of variables among different BNP categories was compared by One-Way ANOVA test, and then Student-Newman-Keuls pairwise comparisons were performed if *P* <0.05. Difference of BNP levels in subjects with normal and abnormal lipids was analyzed by Mann–Whitney *U* test. Univariate association between BNP and continuous variables was expressed as Spearman’s correlation coefficients. Values were transformed to the log base of 10 to obtain standard normal variables if needed. Stepwise multiple linear regression was used to correct the effects of covariates and to test the independent factors. Logistic regression was performed to estimate the OR and corresponding 95 % CI for dyslipidemia. Data were analyzed by software SPSS version 16.0 (SPSS Inc., Chicago, IL, USA). P <0.05 (two-tailed) was considered to be statistically significant for all analysis.

## Results

### The clinical and biochemical characteristics of the study population

The clinical, biochemical and anthropometric characteristics of the study population stratified by BNP quartiles were summarized in Table 1. The subjects were divided into four quartiles according to BNP levels (median values: 7.0, 14.9, 30.6 and 65.5 pg/ml, respectively). The prevalence of diabetes, hypertension, alcohol consumption and smoking was comparable among four groups. TSH, aspartate aminotransferase (AST), gamma-glutamyl transpeptidase (GGT), eGFR, non-HDL and hemoglobin A1c (HbA1c). Compared to the highest BNP quartile (Q4), the lower BNP quartile (Q1 and/or Q2) tended to have younger age, lower Cr and HDL-C, and higher alanine aminotransferase (ALT), BMI, TC, LDL-C and TAG (*P* <0.05). The levels of UA followed a trend of biphasic alteration with the elevation of BNP quartiles. BNP Q3 had significantly lower levels of UA compared to BNP Q1 and Q4 (*P* <0.05). These findings suggest that BNP levels correlate with age, ALT, Cr, BMI, TC, LDL-C, HDL-C, TAG and UA in this elderly population.

**Table 1:** Difference in clinical characteristics of the population stratified by BNP quartiles

Variables	BNP Q1	BNP Q2	BNP Q3	BNP Q4	*P* value
	*n* = 170	*n* = 170	*n* = 170	*n* = 170	
Men, n (%)	101 (59.4)	72 (42.4)	67 (39.4)	94 (55.3)	**<0.0001** ^**a**^
Age, years	68 ± 5	68 ± 5	69 ± 6	70 ± 5	**0.015** ^**b**^
BNP, pg/ml	7.0 (5.5–8.0)	14.9 (11.2–17.3)	30.6 (24.3–37.4)	65.5 (53.9–83.5)	-
BNP range, pg/ml	2.2–9.0	9.1–20.4	20.5–44.4	44.6–99.7	-
BMI, kg/m^2^	23.4 ± 4.1	24.4 ± 4.0	23.1 ± 4.3	22.5 ± 3.3	**0.017** ^**c**^
ALT, IU/L	28.5 ± 19.7	21.2 ± 13.0	18.9 ± 11.5	19.3 ± 21.0	**<0.0001** ^**d**^
AST, IU/L	27.0 ± 14.8	24.0 ± 12.1	22.5 ± 10.1	25.2 ± 20.8	0.232
GGT, IU/L	45.6 ± 58.0	30.1 ± 62.2	32.9 ± 48.6	34.7 ± 43.3	0.259
UA, mg/dL	5.5 ± 1.3	5.2 ± 1.4	5.1 ± 1.5	5.5 ± 1.6	**0.04** ^**e**^
Cr, mg/dL	0.78 ± 0.19	0.74 ± 0.18	0.76 ± 0.24	0.81 ± 0.25	**0.0334** ^**f**^
eGFR (ml/min/1.73 m^2^)	74.3 ± 25.9	73.6 ± 15.4	73.1 ± 19.9	70.2 ± 20.8	0.1860
TC, mg/dL	191.3 ± 37.3	200.1 ± 38.7	188.1 ± 34.2	179.6 ± 38.6	**<0.0001** ^**g**^
LDL-C, mg/dL	115.7 ± 35.0	119.0 ± 37.0	108.5 ± 33.2	107.4 ± 33.7	**0.022** ^**h**^
HDL-C, mg/dL	49.1 ± 11.5	55.7 ± 15.2	53.6 ± 13.8	53.0 ± 16.4	**0.003** ^**i**^
TAG, mg/dL^※^	147.0 (107.3–223.0)	131.0 (91.75–184.0)	116.0 (91.0–170.0)	121.0 (83.0–158.3)	**<0.0001** ^**j**^
non-HDL, mg/dL	142.2 ± 34.6	143.5 ± 37.1	133.8 ± 33.2	125.9 ± 33.3	0.081
TSH, mIU/L^※^	1.6 (1.1–3.2)	1.5 (0.9–2.4)	1.5 (1.1–2.3)	1.5 (0.9–2.9)	0.714
HbA1c,%	6.0 ± 0.8	6.1 ± 1.3	6.1 ± 1.3	6.0 ± 1.1	0.3629
Diabetes, n (%)	49 (28.8)	36 (21.2)	33 (19.4)	38 (22.4)	0.182
Hypertension, n (%)	118 (69.4)	109 (64.1)	113 (66.5)	121 (71.2)	0.516
Current Smoker, n (%)	25 (14.7)	15 (8.8)	16 (9.4)	18 (10.6)	0.296
Current Drinker, n (%)	13 (7.6)	16 (9.4)	9 (5.3)	13 (7.6)	0.552

Data are expressed as mean ± SD for data with normal distribution, or median (25th, 75th percentiles) for data with skewed distribution, or number (percentage) for categorical data. Chi-square test was used to compare categorical variables. The difference of continuous variables was compared using One-Way ANOVA, and then Student-Newman-Keuls pairwise comparisons were performed if *P* <0.05. *P* values less than 0.05 are marked in bold. Note: (1) ※ Analysis was performed using log-transformed data. (2) a. Q1 compared with Q2 and Q3 (*P* = 0.002, <0.0001); Q4 compare with Q2 and Q3 (*P* = 0.0017, 0.003); b. Q4 compared with Q1, Q2 and Q3 (*P* = 0.03, 0.008, 0.073); c. Q2 compared with Q3 and Q4 (*P* = 0.027, 0.002); d. Q1 compared with Q2, Q3 and Q4 (*P* = 0.006, <0.0001, <0.0001); e. Q3 compared with Q1 and Q4 (*P* = 0.025, 0.016); f. Q4 VS Q2, *P* < 0.05; g. Q2 compared with Q3 and Q4 (*P* = 0.010, <0.0001); Q1 compared with Q4 (*P* = 0.015); h. Q2 compared with Q3 and Q4 (*P* = 0.016, 0.009); i. Q1 compared with Q2, Q3 and Q4 (*P* < 0.0001,0.013,0.036); j. Q1 compared with Q2, Q3 and Q4 (*P* = 0.006, <0.0001, <0.0001)

### Association between plasma BNP levels with clinical parameters

Table 2 shows the association of clinical characteristics with plasma BNP levels by using univariable correlation and multiple linear regression analysis. BNP as the dependent variable was logarithmically transformed to approximate normal distribution. In univariable correlation, age was positively correlated with plasma BNP (Spearman’s ρ = 0.361), whereas ALT, GGT, eGFR, TC, TAG and non-HDL were negatively correlated with plasma BNP (ρ = −0.327, −0.175, −0.117, −0.161, −0.184 and −0.170, respectively). In addition, BMI and LDL-C were negatively associated with plasma BNP with marginally statistical significance. After stepwise multiple regression adjusting for covariates (model R^2^ = 0.271, *P* < 0.01), age was still positively associated with plasma BNP (β = 0.051), whereas BMI, eGFR and non-HDL were inversely associated with plasma BNP (β = −0.047, −0.189 and −0.113, respectively). The data indicate that BNP levels, in addition to age, BMI, non-HDL and eGFR, were significantly associated with nonfasting lipid parameters.

**Table 2:** Linear and multiple regression analysis of association between metabolic parameters and BNP levels (Log BNP) in the subjects

Variables	Univariate correlation		Multivariate regression	
	ρ coefficient	*P* value	β coefficient	*P* value
Gender (men,1; women, 0)	−0.028	0.602		
Age (years)	0.361	**<0.001**	0.051	**<0.001**
BMI (kg/m^2^)	−0.128	0.054	−0.047	**0.001**
ALT (IU/L)	−0.327	**<0.001**		
AST (IU/L)	−0.089	0.093		
GGT (IU/L)	−0.175	**0.001**		
UA (mg/dL)	0.035	0.563		
Cr (mg/dL)	0.054	0.306		
eGFR (ml/min/1.73 m^2^)	−0.117	**0.027**	−0.189	**0.005**
TC (mg/dL)	−0.161	**0.007**		
LDL-C (mg/dL)	−0.119	0.054		
HDL-C (mg/dL)	0.050	0.403		
Log TAG (mg/dL)	−0.184	**0.001**		
non-HDL (mg/dL)	−0.170	**0.008**	−0.113	**<0.001**
Log TSH (mIU/L)	−0.021	0.650		
HbA1c (%)	0.125	0.321		
Diabetes (yes = 1; no = 0)	−0.045	0.481		
Hypertension (yes = 1; no = 0)	0.012	0.621		
Smoking (yes = 1; no = 0)	0.008	0.724		
Drinking (yes = 1; no = 0)	0.005	0.565		

*P* values less than 0.05 are marked in bold

### Dyslipidemia prevalence in individuals with different BNP quartiles, and BNP levels in subjects with normal or abnormal lipid levels

**Fig. 1:**
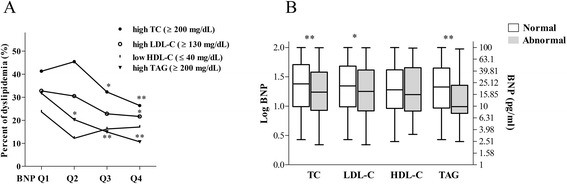
**a** The prevalence of abnormal lipid profiles stratified by different BNP quartiles (*, *P* <0.01 vs. Q1; **, *P* <0.001 vs. Q1). The cut-off points of dyslipidemia were defined as described in the methods. Chi-square test was used to compare the difference. **b** The difference of BNP levels between normal and abnormal groups according to lipid profiles. The lines denote the median, the boxes represent interquartile range, and the bars indicate the lowest or highest BNP levels. Boxes in grey denote patients with abnormal lipid profiles, whereas open boxes represent patients with normal lipid profiles (Mann–Whitney *U* test was used. *, *P* <0.01 vs. normal group; **, *P* <0.001 vs. normal group). Note that numbers on the right vertical axis are corresponding to log-transformed values on the left axis

We next sought to explore the potential relationships between BNP levels and dyslipidemia. As shown in Fig. 1a, the prevalence of dyslipidemia gradually reduced with the increase of BNP levels, including high TC, high TAG and high LDL-C (*, *P* <0.01; **, *P* <0.001; compared to BNP Q1). Figure 1b shows the BNP levels in normal and abnormal lipid groups. The medians of BNP were significantly lower in subjects with abnormal TC, LDL and TAG than those with normal respective lipids (*, *P* <0.01; **, *P* <0.001). These findings demonstrate that higher BNP levels negatively correlated with the prevalence of nonfasting dyslipidemia.

### Subjects with lower BNP quartiles (Q1-3) tend to have raised risks for nonfasting dyslipidemia compared to the highest BNP quartile (Q4)

**Fig. 2:**
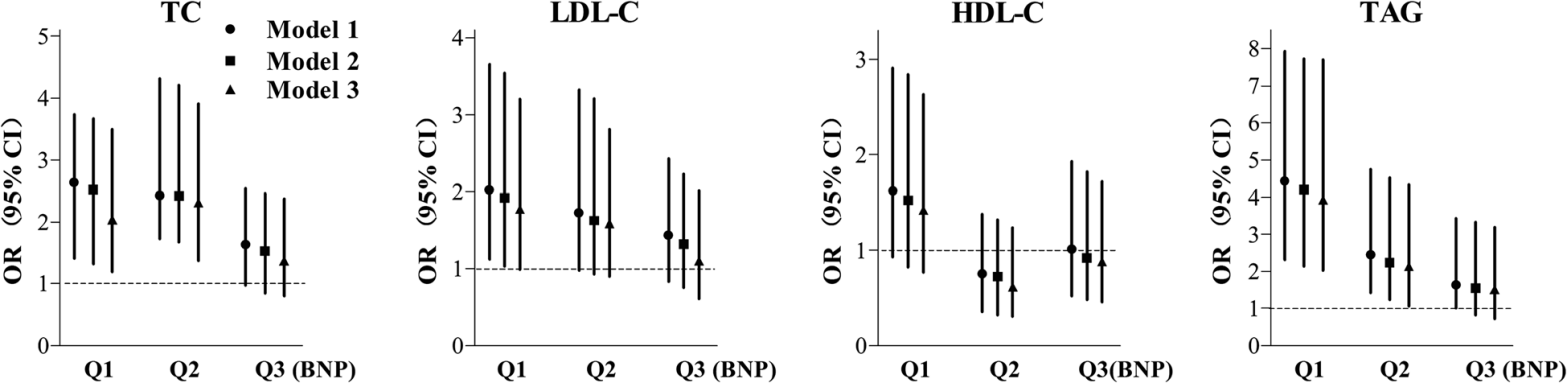
Logistic regression to evaluate the OR and 95 % CI for dyslipidemia in BNP quartiles (Q1, Q2, Q3) compared to BNP Q4. Model 1: adjusted for smoking, hypertension, drinking, diabetes, HbA1c, TSH, ALT, AST, GGT and UA; Model 2: Model 1 + eGFR; Model 3: Model 2 + age + BMI

Adjusted odds ratios (OR) with associated 95 % confidence intervals (CI) of nonfasting dyslipidemia in the BNP Q1-3 were shown in Fig. 2, with BNP Q4 as reference. In model 1 (adjusted for smoking, hypertension, drinking, diabetes, HbA1c, TSH, ALT, AST, GGT and UA), the risks of hypercholesterolemia, hyper-LDL-emia and hypertriglyceridemia were significantly elevated in the BNP Q1 group, while the risks for hypertriglyceridemia was progressively reduced in BNP Q2 and Q3 group. In model 2 (additionally adjusted for eGFR) and model 3 (further adjustment for age and BMI), the results were similar to model 1. In model 3, individuals in the BNP Q1 and Q2 quartiles had increased risks for nonfasting dyslipidemia (OR [95 % CI] for BNP Q1: TC 2.042 [1.192–3.500], *P* = 0.009; Q2: TC 2.316 [1.372–3.911], *P* = 0.002; BNP Q1: TAG 3.938 [2.013–7.702], *P* < 0.0001; Q2: TAG 2.134 [1.051–4.336], *P* = 0.036). The OR for hyper-LDL-emia in the BNP Q1 quartile reached marginally statistical significance (OR [95 % CI]: 1.778 [0.986–3.203], *P* = 0.056). These findings showed that subjects with higher BNP levels tended to have reduced risks for nonfasting dyslipidemia, especially for hypercholesterolemia and hypertriglyceridemia.

## Discussion

In this cross-sectional study, we found that nonfasting dyslipidemia in the elderly Japanese population negatively correlated with BNP levels within the physiological range. The elderly people with the lowest BNP quartile were prone to have obviously elevated risks for nonfasting hypercholesterolemia and hypertriglyceridemia, as opposed to subjects with higher BNP quartiles. Although the correlation between BNP and lipid profiles was found but was not potent enough in linear analysis model, results from multiple regression analysis further supported this association independent of several clinical variables.

N-terminal of the prohormone brain natriuretic peptide (NT-proBNP) which is equimolarly released into circulation with BNP, has also been used widely as a surrogate biomarker for BNP. Recently, the levels of fasting LDL-C and TAG have been demonstrated to be inversely associated with NT-proBNP and followed a biphasic pattern, with the turning point at 100 pg/ml [21]. The linear associations between lipids and NT-proBNP became not obvious for those whose NT-proBNP levels were higher than 100 pg/ml. This study also established a positive correlation of HDL-C level with NT-proBNP, which is a little different from our results. Although the linear trend between HDL-C and BNP was not observed in the current population, subjects with BNP Q1 had markedly reduced HDL-C than subjects with BNP Q2-4 (Table 1). Nevertheless, both studies have consistently confirmed increased risks of dyslipidemia for individuals with low BNP levels regardless of the ethnicity or fasting status.

The mechanisms for the association between BNP levels and lipid metabolism can be ascribed to several reasons. BNP levels are determined by the dynamic balance of production and clearance. Firstly, several humoral factors are involved in the modulation of BNP secretion, including glucagon-like peptide-1 (GLP-1) [1]. GLP-1 is well-recognized as an intestinal hormone with significantly reduced levels in obese patients [22]. GLP-1 directly promotes the secretion of ANP in atrial cardiomyocytes through GLP-1 receptor [23]. Although it remains unknown whether the similar phenomenon exists in BNP secretion, another clinical study observed that ANP and BNP both were elevated after GLP-1 analogue treatment [24]. Chronic inflammation triggered by relative adipocyte hypoxia is a hallmark in metabolic disorders [25]. As a well-known factor involved in insulin resistance, tumor necrosis factor-α (TNF-α) also impairs GLP-1 secretion from the intestinal L-cells [26]. This is in accord with the clinical observation that patients with fatty liver disease or obesity have low levels of GLP-1 [27, 28]. Secondly, plasma BNP is cleared through receptor-mediated degradation and extracellular protease-dependent pathways. Natriuretic peptide clearance receptor (NPR-C) is a pseudo-receptor which binds the BNP for internalization and degradation. Increased NPR-C in the adipose tissue accelerates the clearance of BNP in the obese individuals [29, 30]. In addition, insulin-degrading enzyme (IDE) and dipeptidyl peptidase-4 (DPP-4) have been demonstrated to inactivate BNP in a protease-dependent manner [31, 32]. Recently, it has been shown that the activity of IDE in the liver is enhanced in a diet-induced obesity model [33]. DPP-4 hyperactivity has been observed in morbidly obese patients [34, 35]. Thus, multiple mechanisms are responsible for the BNP handicap phenomenon in metabolic syndrome. Thirdly, BNP is dependent on renal function for clearance [36]. So, it is reasonable that age-dependent BNP elevation may be partly due to the decline of eGFR with aging. The age-related elevation of BNP might be a protective mechanism for favorable lipid metabolism. The U-shaped alteration of UA levels from the lowest to highest BNP quartiles (Q1-Q4) may be the consequence of reduced metabolic syndrome together with elevated eGFR.

In our study, subjects within BNP Q1 had higher ALT levels than individuals with BNP Q2-4, which may be caused by increased prevalence of fatty liver diseases. Similarly, the mean BMI in BNP Q2 group was markedly higher than BNP Q3-4. From the observation that BNP is inversely related with visceral fat and liver fat [12], we may extrapolate that high prevalence of metabolic hepatic abnormalities and dyslipidemia in people with low levels of BNP are in part caused by ectopic fat accumulation. Interestingly, BNP also functions to increase the feeling of satiety. People with low levels of BNP are predisposed to have increased appetite which results in obesity [37]. Higher ALT and insulin resistance have long been considered as a risk factor for high TAG levels [38]. In addition, BNP levels have been shown to be negatively correlated with insulin resistance irrespective of obesity [39]. Insulin resistance in the liver not only promotes the *de novo* hepatic lipogenesis but also reduces the degradation of TAG-enriched lipoproteins, leading to both fasting and postprandial hypertriglyceridemia [40]. Insulin sensitivity index has repeatedly been documented to be associated with increased cholesterol synthesis in a hyperglycemia-independent manner [41, 42].

Traditionally, lipid levels have been determined in the fasting state mainly due to the reduced variability than random sampling. The evidences from a large number of epidemiological studies repeatedly support the risk-predictive values of nonfasting lipids, especially nonfasting TAG, for CVD [18]. Individuals with hyperglyceridemia are prone to produce strongly atherogenic lipoproteins, such as cholesterol-rich remnant-like lipoproteins and small dense LDL particles [43]. The finding in this study that significantly higher prevalence of hyperglyceridemia links with the lowest BNP Q1 quartile (2.2–9.9 pg/ml) is consistent with another recently published observation [21]. Therefore, individuals with lower BNP levels may have increased risk for CVD events regardless of the races, age, gender and BMI. As mentioned earlier, higher levels of BNP correlates with increased vascular events and all-cause mortality in Justification for the Use of Statins in Prevention: An Intervention Trial Evaluating Rosuvastatin (JUPITER) [3], which is inconsistent with our observation. This discrepancy might be caused by the eligible participants involved in JUPITER who had LDL-C <130 mg/dL. Therefore, the study has excluded people with lower BNP levels which often link with dyslipidemia. It should be acknowledged that people with higher BNP often have increased risks for cardiovascular events, which includes older age, myocardial injuries and elevated eGFR. Thus, our study doesn’t imply that higher BNP correlates with better clinical outcome.

However, several limitations exist in our current study. Firstly, data on waist circumference which reflect visceral obesity are lacking. Secondly, insulin resistance which is a predisposing factor for dyslipidemia may be associated with BNP levels. The insulin resistance index and glucose metabolism are unknown in the study. Thirdly, our cross-sectional observation is unable to establish potential cause-effect relationship between BNP levels and dyslipidemia. In addition, the sample size of the population is not large enough. Thus, our findings require further studies to identify the relationship between plasma BNP levels and lipid profiles.

## Conclusions

In conclusion, the current cross-sectional study provides further evidence about the metabolic risks of low BNP levels in the elderly. Elderly individuals with lower BNP levels tend to have higher prevalence of nonfasting dyslipidemia. Thus, it warrants further investigation whether BNP could serve as a biomarker or have therapeutic implications for dyslipidemia in the elderly people.

## Abbreviations

ANP: atrial natriuretic peptide
BNP: B-type natriuretic peptide
CNP: C-type natriuretic peptide
BMI: body mass index
CVD: cardiovascular diseases
TAG: triglyceride
TC: total cholesterol
LDL-C: low-density lipoprotein cholesterol
HDL-C: high-density lipoprotein cholesterol
TSH: thyroid stimulating hormone
AST: aspartate aminotransferase
GGT: gamma-glutamyl transpeptidase
eGFR: estimated glomerular filtration rate
HbA1c: hemoglobin A1c
Cr: creatinine
ALT: alanine aminotransferase
UA: uric acid
OR: odds ratios
CI: confidence intervals
NT-proBNP: N-terminal of the prohormone brain natriuretic peptide
GLP-1: glucagon-like peptide-1
TNF-α: tumor necrosis factor-α
NPR-C: natriuretic peptide clearance receptor
IDE: insulin-degrading enzyme
DPP-4: dipeptidyl peptidase-4
JUPITER: justification for the use of statins in prevention: an intervention trial evaluating rosuvastatin
SD: standard deviation
ANOVA: analysis of variance
